# Thermomechanical Properties of Neutron Irradiated Al_3_Hf-Al Thermal Neutron Absorber Materials

**DOI:** 10.3390/ma16165518

**Published:** 2023-08-08

**Authors:** Donna Post Guillen, Mychailo B. Toloczko, Ramprashad Prabhakaran, Yuanyuan Zhu, Yu Lu, Yaqiao Wu

**Affiliations:** 1 Idaho National Laboratory, 995 University Blvd., Idaho Falls, ID 83401, USA; 2 Pacific Northwest National Laboratory, 902 Battelle Blvd., Richland, WA 99354, USA; mychailo.toloczko@pnnl.gov (M.B.T.); ramprashad.prabhakaran@pnnl.gov (R.P.); 3 Department of Materials Science & Engineering, University of Connecticut, 25 King Hill Road, Storrs, CT 06269, USA; yuanyuan.2.zhu@uconn.edu; 4 Center for Advanced Energy Studies, Boise State University, 997 MK Simpson Blvd., Idaho Falls, ID 83401, USA; yulu@boisestate.edu (Y.L.); yaqiaowu@boisestate.edu (Y.W.)

**Keywords:** intermetallic, neutron irradiation, microindentation hardness, tensile testing, thermal expansion, neutron absorber, nanoindentation, Al_3_Hf-Al

## Abstract

A thermal neutron absorber material composed of Al_3_Hf particles in an aluminum matrix is under development for the Advanced Test Reactor. This metal matrix composite was fabricated via hot pressing of high-purity aluminum and micrometer-size Al_3_Hf powders at volume fractions of 20.0, 28.4, and 36.5%. Room temperature tensile and hardness testing of unirradiated specimens revealed a linear relationship between volume fraction and strength, while the tensile data showed a strong decrease in elongation between the 20 and 36.5% volume fraction materials. Tensile tests conducted at 200 °C on unirradiated material revealed similar trends. Evaluations were then conducted on specimens irradiated at 66 to 75 °C to four dose levels ranging from approximately 1 to 4 dpa. Tensile properties exhibited the typical increase in strength and decrease in ductility with dose that are common for metallic materials irradiated at ≤0.4T_m_. Hardness also increased with neutron dose. The difference in strength between the three different volume fraction materials was roughly constant as the dose increased. Nanoindentation measurements of Al_3_Hf particles in the 28.4 vol% material showed the expected trend of increased hardness with irradiation dose. Transmission electron microscopy revealed oxygen at the interface between the Al_3_Hf particles and aluminum matrix in the irradiated material. Scanning electron microscopy of the exterior surface of tensile tested specimens revealed that deformation of the material occurs via plastic deformation of the Al matrix, cracking of the Al_3_Hf particles, and to a lesser extent, tearing of the matrix away from the particles. The fracture surface of an irradiated 28.4 vol% specimen showed failure by brittle fracture in the particles and ductile tearing of the aluminum matrix with no loss of cohesion between the particles and matrix. The coefficient of thermal expansion decreased upon irradiation, with a maximum change of −6.3% for the annealed irradiated 36.5 vol% specimen.

## 1. Introduction

Fast spectrum neutron irradiation environments are necessary to support materials and fuels research and development for the next generation of fast nuclear reactors [1]. Until a fast flux test reactor is available, efforts to develop advanced nuclear fuel and materials for future nuclear power plants in the U.S. have focused on attaining comparable irradiation conditions within existing thermal or mixed spectrum reactors by locally modifying their neutron spectra [2,3]. One such approach, studied by researchers at the Idaho National Laboratory for use within the Advanced Test Reactor (ATR), is known as the boosted fast flux loop. This design proposes surrounding the northwest lobe within the reactor core with a Hf-Al thermal neutron absorber blanket (or neutron filter) to increase the local fast-to-thermal ratio (FTR) [4]. Adding uranium silicide booster fuel surrounding the absorber blanket would augment the local fast neutron flux [4].

The selection of materials for the neutron absorber blanket focused on the binary hafnium-aluminum system. Hafnium has a large cross section for thermal neutron capture while aluminum has a high thermal conductivity. This is a desirable selection of elements that should allow the heat generated by neutron capture to be effectively conducted to coolant channels. The aluminum-hafnium phase diagram shows that, similar to other early transition metal aluminum alloys, it has extremely low solid solubility of the two components, with intermetallic phases forming across virtually all compositions [5] at temperatures below 650 °C where this material would be used. The maximum solubility of Hf in solid Al is 0.186 at%. [6,7,8]. A hafnium and aluminum melt will solidify into several possible intermetallic phases depending on the elemental ratios in the melt and the cooling conditions [9]. Rather than attempting to make alloys from the melt, a decision was made to fabricate alloys by hot pressing Al_3_Hf powders in an Al matrix.

Neutronics calculations indicate that an absorber block material comprised of an aluminum matrix composite containing 28.4 vol% (volume percent) Al_3_Hf intermetallic particles corresponding to 7.00 at% (atom percent) hafnium surrounded by three rings of uranium silicide booster fuel yields a fast flux of 10^19^ n/m^2^/s and an FTR of 40, while maintaining all components below their maximum temperature limits [10,11]. Moreover, the Al_3_Hf phase is stable up to the melting point of aluminum. Hafnium has been shown to be resistant to corrosion in steam and water up to 315–399 °C [12], a property that has been reported to not be adversely affected by neutron irradiation [13]. This material can be classified as a metal matrix composite (MMC).

One goal of this research is to evaluate the tensile properties of unirradiated and irradiated Al_3_Hf-Al MMCs. Although the neutron absorber block is not planned for use in structural components, maintaining acceptable mechanical properties is important since spent absorber blankets will need to be periodically inspected and replaced with fresh ones [14]. The results presented here provide mechanical and thermal properties needed for component design.

Understanding the effects of irradiation on the tensile properties of this MMC requires knowledge of the irradiation effects on both the Al matrix and the Al_3_Hf intermetallic particles. For Al, the mechanisms of radiation damage and their effects on material properties are well understood and depend predominately on the neutron spectrum, thermal and fast fluences, irradiation temperature, and the concentrations and types of solute or impurity elements [15]. Through transmutation reactions of Al, fast neutrons produce mostly hydrogen, helium, sodium, and magnesium, while thermal neutrons produce mostly silicon [15]. If thermal fluences exceed ~10^25^ n/m^2^ [16], Si will precipitate, causing an increase in tensile strength and a reduction in ductility. Void swelling will also occur as the dose increases [17,18]. Farrell et al. [18,19] found that the irradiation of lower-purity Al alloys results in significantly less cavity formation than high-purity Al, a result largely attributed to the reduction in vacancy mobility by binding with solute elements [19].

Group III–V transitional metal trialuminides (denoted Al_3_M) have been extensively investigated over the past several decades as thermally stable precipitate strengtheners [20,21]. These intermetallics are thermally stable in an Al matrix [22]. For particles of a sufficiently small size to act as barriers to dislocation movement within the matrix, the high thermal stability limits precipitate growth, thus limiting the loss of dislocation barriers [23]. The resistance to high-temperature particle coarsening is also improved by the low lattice mismatch between the Al_3_M intermetallic and the Al matrix [24,25]. Several investigations have sought to further reduce this lattice mismatch by introducing additional transition metal alloy components [21,23,26]. These features result in stable mechanical properties at temperatures needed to serve as a thermal neutron absorber in the ATR.

Tensile testing, microhardness, nanoindentation, scanning electron microscopy (SEM), and transmission electron microscopy (TEM) were performed to assess how neutron irradiation affected the mechanical properties and microstructure of particles and matrix. Thermal expansion measurements were also conducted.

## 2. Experimental Methods

### 2.1. Materials, Specimens, and Irradiation Conditions

The intermetallic component (Al_3_Hf) of the MMC was formed by a centrifugal casting process. Based on preliminary studies, hafnium bar stock and laser-welded aluminum granules were placed together at a ratio of 69 wt% to 31 wt%, respectively, in a crucible for casting. The casting temperature was ~1450 °C, and water quenching was performed directly after casting. In general, the D022 and/or D023 crystal structures of Al_3_M, while responsible for their high strengths, also cause them to be brittle near ambient temperature [12]. The castings were-ground into powder and sieved with an ASTM No. 400 mesh to retain particles smaller than 38 µm. A larger number of smaller particles is preferred over fewer large particles to provide a more even distribution of heat (due to neutron absorption) throughout the material. The intermetallic particles were mixed with the required amount of aluminum powder (Alcoa 101, 99.5Al-0.25Si-0.15Fe) to produce MMCs with 20.0, 28.4, and 36.5 vol% Al_3_Hf, which corresponds to 4.95, 7.00, and 9.00 at% Hf. The 28.4 vol% material was found to be optimum from a neutronics and thermal standpoint [6,7]. The other two volume percentages were selected to bound the optimum composition.

**Figure 1:**
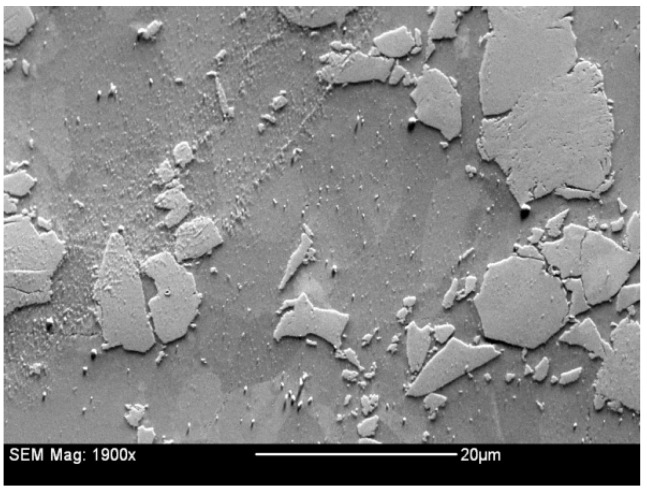
A SEM secondary electron image of the 28.4 vol% Al_3_Hf-Al material.

Prior proof-of-principle studies concluded that cold pressing or pressureless sintering were incapable of providing materials with sufficient machinability. Therefore, a hot uniaxial pressing process was used to consolidate the materials wherein a powdered sample was heated in a vacuum furnace to 585 °C and then subjected to a prescribed pressure of 1.103 MPa to densify the material into pucks. Specimens were subsequently fabricated from the pucks via electrical discharge machining. An SEM secondary electron image of the 28.4 vol% hot pressed material shown in Figure 1 shows Al_3_Hf particles in the aluminum matrix.

**Figure 2:**
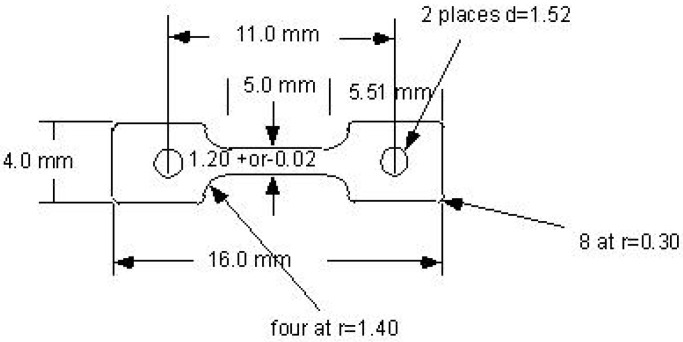
S1 tensile geometry used for this study. Specimens are 1.0 mm thick.

One-millimeter-thick tensile specimens of the S1 geometry (Figure 2) were machined from the pucks to use for tensile property and hardness characterization. The S1 geometry is frequently used for studies of irradiation effects on tensile properties.

The specimens were irradiated in the ATR with the estimated irradiation temperatures and doses listed in Table 1 [27,28]. The irradiation temperatures that were computed from finite-element analysis using volumetric heat rates from a Monte Carlo N-Particle physics analysis [29] ranged from 66 to 75 °C [27], and all three materials received approximately the same doses [28]. The fast-to-thermal ratio during the ATR experiment ranged from 0.35 to 0.55. This uniformity in irradiation environment among the three materials facilitates determination of irradiation effects on the properties. Table 2 shows the density of Al_3_Hf-Al samples as a function of volume fraction of Al_3_Hf and the weight percent of the Al, Hf and Zr. Zirconium is present as an impurity in the Hf metal. Density was measured by the Archimedes method, and elemental composition was determined by SEM/Energy Dispersive X-ray Spectroscopy (EDS).

### 2.2. Test Methods

#### 2.2.1. Microhardness Tests

After irradiation, one side of each tensile specimen was polished to a 1 µm surface finish by mounting the specimens against pucks using hot glue and performing a series of hand polishing steps that concluded with 1 µm diamond paste on a soft cloth. To prevent rocking of the pucks during polishing, and thereby avoiding uneven thickness reduction, aluminum strips of the same thickness as the tensile specimens were mounted around the perimeter of the pucks. This process was verified first on unirradiated control specimens before proceeding to the irradiated specimens. Among all specimens, the greatest variation in thickness observed along the gauge length was 0.025 mm, which for a nominal 1 mm thick specimen represents a 2.5% variation in cross-sectional area. Most specimens had a thickness variation of ≤0.012 mm (≤1.2% variation in cross-sectional area).

**Figure 3:**
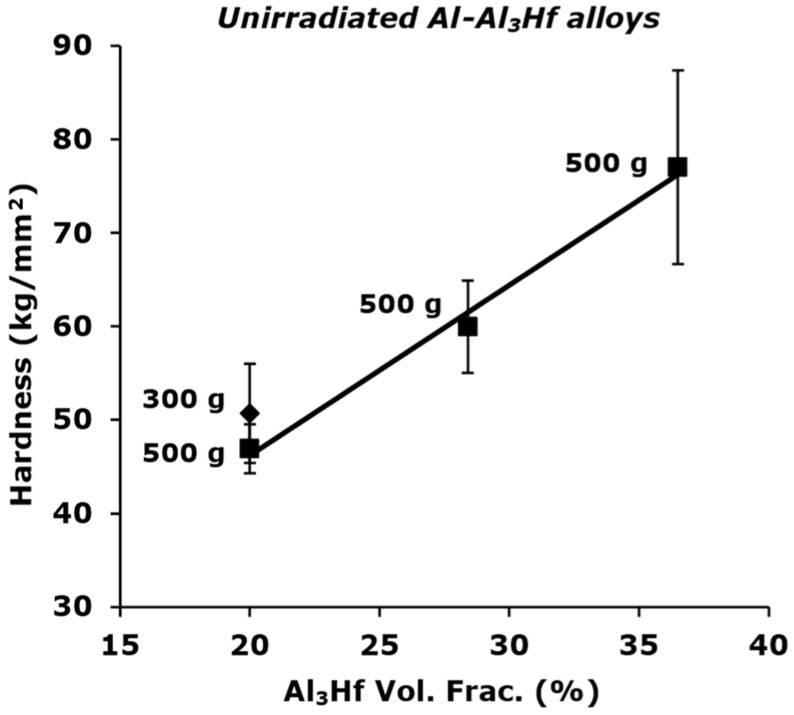
Vickers microhardness values of unirradiated Al_3_Hf-Al samples (20 vol% Al_3_Hf) with a comparison between 300 g and 500 g loads. Error bars are the standard deviation of each measurement set.

Prior to tensile testing, Vickers microhardness measurements were performed on both tabs of each specimen at room temperature (RT). To determine an appropriate load for the hardness testing, a series of tests were conducted on the three different materials in the unirradiated condition. For the 28.4 and 36.5 vol% materials, a 500 g load produced 110–125 µm indentations that sampled several grains and had a depth much less than one-tenth of the specimen thickness (minimum ASTM E92 requirement) [30]. However, because there was some concern that the 20.0 vol% material would require a lighter load to be within the ASTM specification, both 300 g and 500 g loads were assessed. The indentations for the 500 g load were found to be only slightly larger at ~140 µm, making them a viable size. Figure 3 shows that only a small difference in hardness was observed for the 300 g and 500 g loads on the 20.0 vol% material, and the decision was made to use a 500 g load for all irradiation conditions and specimens. The trend line is fitted to the 500 g data. Ten indentations were performed on both tabs of each specimen and combined to make a dataset.

#### 2.2.2. Tensile Tests

This material is being developed in support of nuclear reactor applications where the expected operating temperature of the absorber component is ~110–225 °C [4]. Neutron irradiations of the materials were performed at ~70 °C, which is below the expected component operating temperature and is likely to cause more hardening and loss of ductility than for irradiations performed at 110–225 °C. Tensile tests were conducted at both RT and 200 °C for the unirradiated specimens, but only at 200 °C for the irradiated specimens due to their limited number. Two hundred degree celsius was selected because it lies within the operating temperature range of the component and because exposure of the specimens to this temperature during heating may slightly anneal out some of the lower irradiation temperature damage prior to performing a tensile test, potentially making the observed tensile properties more representative of material irradiated between 110 and 225 °C.

**Figure 4:**
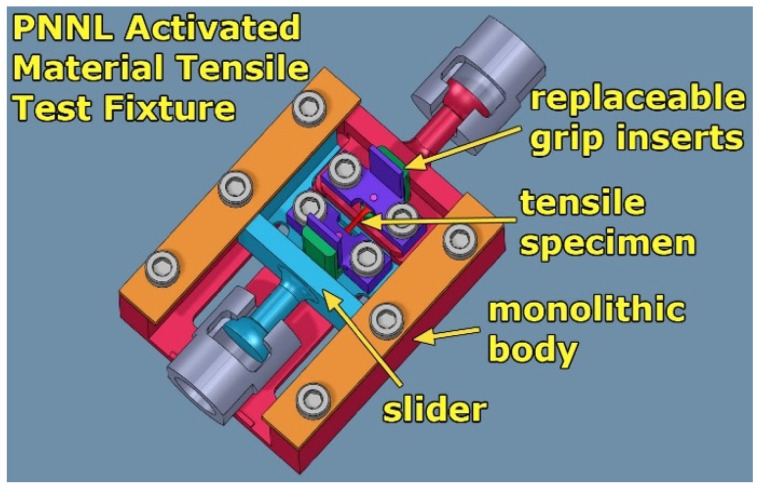
Activated material tensile test fixture for miniature tensile specimens.

Tensile tests were performed using the fixture illustrated in Figure 4 that allows only axial straining of the specimens and prevents damage to the specimen by twisting and bending. Alignment of the specimen with the pulling direction of the fixture is achieved by using pins that go through the hole in both tabs of the tensile specimens. A carriage for the fixture applies ~0.5 kg of spring preloading to align the specimen before the grips are tightened.

Tensile tests were conducted in an Instron 8801 servohydraulic frame. The crosshead speed was selected to provide a 1 × 10^−4^ s^−1^ strain rate assuming a completely stiff load train. As is commonly performed for miniature tensile specimens of this size, the strain was estimated from the actuator displacement. Heating was accomplished using a three-zone clamshell air furnace. Tests were started a few minutes after reaching the target temperature. Load, specimen gauge dimensions, and test temperature were measured using equipment verified against standards with NIST traceable certifications. Tensile properties reported here are engineering values.

Only one tensile specimen per combination of alloy and irradiation condition was available, hence no statistical data could be obtained on the tensile properties. Strength measurement uncertainties that can be quantified include variations in cross section along the length of the gauge region, the accuracy of the micrometer used to measure the gauge thickness and width, and the accuracy of the load cell. These are cumulative values that add up to ~5% uncertainty. Judgement used in determining the strength values from the plots is another source of uncertainty, but unlike the others, it cannot be easily quantified. When analyzing the tensile results, it is also important to recognize that the effect of irradiation on the properties of a material is often strongly dependent on irradiation temperature, and unusual trends in the data may be due to uncertainty in this value at each irradiation dose. Uncertainty in elongation measurements has the same dependencies except that accuracy of actuator movement replaces uncertainty in load cell accuracy.

#### 2.2.3. Dilatometry

Dilatometry was performed using a Netzsch 402C (NETZSCH-Gerätebau GmbH, Selb, Germany) horizontal push-rod dilatometer that measures linear sample displacement during programmed heating. Thermal expansion was performed on specimens in a 5 mm × 5 mm rod geometry. Unirradiated and irradiated specimens with 20.0, 28.4, 36.5 Al_3_Hf vol% and an unirradiated specimen with 100 vol% Al_3_Hf were tested. Table 3 lists the irradiation conditions for the specimens used for the thermal expansion measurements. The table includes the Al_3_Hf vol%, cycle average irradiation temperature, and the total dose. These specimens were irradiated for four cycles in the ATR which equates to 3984.6 MWd and a fluence of 12.02 × 10^25^ n/m^2^.

#### 2.2.4. Microscopy

Examinations of the fractured tensile bars focused on understanding the deformation mode and defect production in the matrix that may affect thermal conductivity and mechanical strength. SEM was used to examine the microstructure at different locations near the fracture surface by taking standard SEM micrographs at different magnifications at several locations on each sample (i.e., low through high magnification to observe various length scales of features, and to confirm homogeneity across the sample).

TEM was performed to understand the strengthening mechanism within the grains. High resolution imaging of the Al matrix was also accomplished using TEM. It was necessary to polish the surface oxide layer before performing a focused ion beam (FIB) lift-out. The surface oxide layer is a consequence of using water to remove radioactive contamination from the specimens introduced during disassembly of the capsules in the hot cell.

An FEI (now ThermoFisher) Quanta 3D dual-beam FIB/SEM was used to prepare the TEM lamellae by using the lift-out technique. Both sides of the lamella were milled with 2 kV Ga+ as a final step to minimize the damage from FIB. Then, the lamella surfaces were cleaned using a Fischione Model 1040 Nanomill with a low beam energy of 600 eV, to further remove the Ga+ damage layers resulting from the FIB process. TEM characterization was performed with a FEI Tecnai G2 F30 STEM. Bright-Field (BF) TEM images were acquired to visualize the coherency of the interface between the particles and the matrix. EDS was applied to study the chemical composition evolution across the interface. On-zone axis BF and Z-contrast STEM images were used to explore the interface between the matrix and the Al_3_Hf dispersion.

#### 2.2.5. Nanoindentation

In addition to studying the matrix microstructure near the Al_3_Hf particles, nanoindentation was used to investigate whether irradiation is making the particles more susceptible to fracture. Nanoindentation was performed on an unirradiated specimen and neutron irradiated material from KGT-1404 using a Hysitron (now Bruker) TI-950 Triboindenter with a diamond Berkovich tip. The surfaces were polished to remove surface oxidation. The unirradiated specimen was polished with sandpaper up to 1200 grit then a diamond suspension used for the final finish. The irradiated samples were jet-polished with 90% methanol and 10% nitric acid (with a concentration of 69–70%) to avoid the use of water. Trials were conducted to determine the proper indentation depth since if the indents are too shallow the results can be affected by the surface finish, whereas if the indents are too deep the results can be affected by the aluminum matrix beneath the particle. The hardness measurements were found to be stable between depths of 130 to 160 nm; therefore, a depth of 150 nm was selected for the measurements. A total of 56 indents were performed using a 7 × 8 rectangular array on each sample. Each indent was separated by 7 μm to avoid the influence from the surrounding indents. Indentation was performed using displacement control, with displacement set to 150 nm. The time frame used for each indentation was 5 s loading, 2 s dwell, and 5 s unloading. After nanoindentation, a FEI (now ThermoFisher) Quanta 3D dual-beam FIB/SEM was used to locate and image the indents. By inspecting the SEM images, it was possible to distinguish the indents that fall on either the aluminum matrix or Al_3_Hf particles.

## 3. Results and Discussion

### 3.1. Hardness Measurements

Microhardness measurements were made to assess the hardness of the bulk structure. Nanoindentation was performed to measure the hardness of the Al_3_Hf particles. Both sets of measurements were made on irradiated and unirradiated specimens to assess the effect of neutron irradiation.

#### 3.1.1. Microhardness

Vickers microhardness values of MMCs are shown in Table 4. For the unirradiated material, a roughly linear trend between hardness and volume fraction is indicated by the data, with an approximate 1.6× increase in the hardness between 20.0% and 36.5% specimens. A consistent relationship between hardness and uniaxial yield strength has been observed for a variety of metallic materials [31,32], and thus hardness testing is often used as an indicator of yield strength change in materials at room temperature. Yield strength change measured in units of megapascals (MPa) is approximately equal to 3× the change in hardness in units of kg/mm^2^ [31]. The hardness data, therefore, suggest a ~90 MPa increase in yield strength between 20 and 36.5 vol%, showing that the Al_3_Hf particles contribute substantially to an increase in strength.

**Figure 5:**
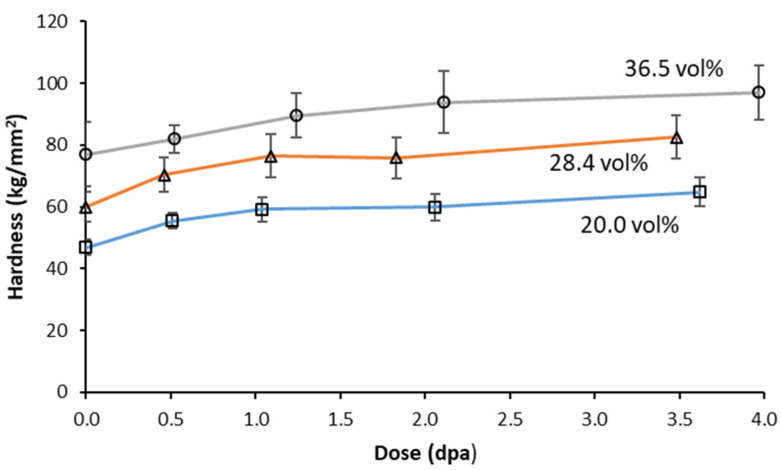
Microhardness of the three MMCs as a function of dose.

Hardness values of the three MMCs are plotted as a function of dose in Figure 5. The data presented are an average of 20 indentations, with error bars showing the standard deviation (SD) of those 20 measurements. The hardness increased with dose and appears to trend towards a plateau value as is typical for metallic materials irradiated at <0.4T_m_, (where T_m_ is the melting temperature) [33,34,35]. The dose after which no further hardening occurs depends on the material and irradiation conditions, and for these materials and irradiation conditions, the majority of the hardening occurs within the first 2 dpa. Interestingly, the three different MMCs underwent almost the same amount of hardening as a function of dose, suggesting that the hardening is not strongly tied to the alteration of the Al_3_Hf dispersion but instead may be due to matrix hardening effects that operate independently of the dispersion. This trend was also observed by Guillen and Harris [36] for the thermal conductivity of the irradiated material.

**Figure 6:**
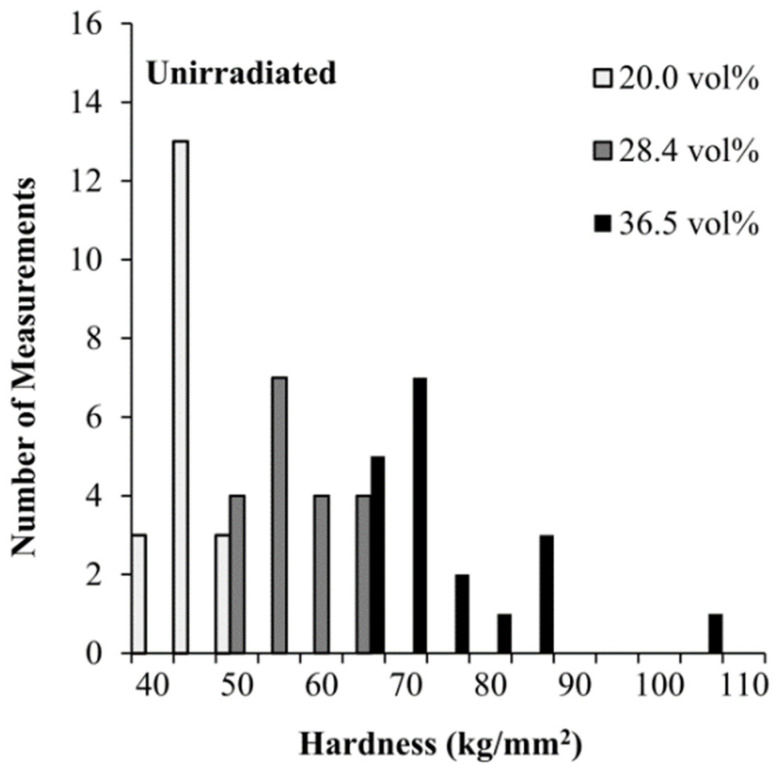
Microhardness frequency distributions for the three MMC variants.

Another trend in the data is an increase in the scatter of the hardness data with increasing Al_3_Hf volume fraction. These microhardness indentations are sufficiently small to be capable of falling in regions either with or without Al_3_Hf particles, and the probability of the indenter landing either partially or completely on an Al_3_Hf particle correlates with the Al_3_Hf volume fraction. Figure 6 shows that as the vol% increases, not only does the mode of the hardness distribution increase, but also the spread in the hardness measurements increases, manifesting as a tail in the high end of the hardness range for the 36.5% volume fraction material. This tail likely represents the hardness indenter encountering increasingly harder regions from denser groupings of the Al_3_Hf particles.

#### 3.1.2. Nanohardness

**Figure 7:**
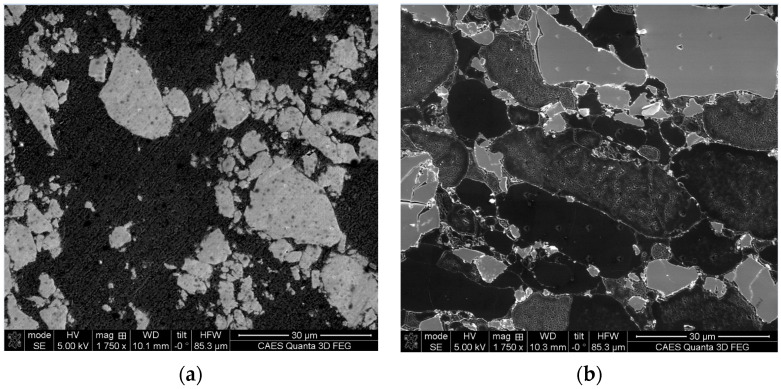
Microstructure of (**a**) 36.5 vol% unirradiated, and (**b**) 28.4 vol% neutron irradiated specimens used for nanoindentation.

The nanohardness of the Al_3_Hf particles was measured on an unirradiated sample and a neutron irradiated sample (KGT-1404). An unirradiated 36.5 vol% specimen was selected for nanoindentation since the probability of an indent landing on a particle is higher than for the lower vol% MMCs. Table 5 lists the average nanohardness and SD values measured on the microstructures shown in Figure 7. Slightly higher hardness was obtained for the Al_3_Hf particles after irradiation.

### 3.2. Tensile Properties

**Figure 8:**
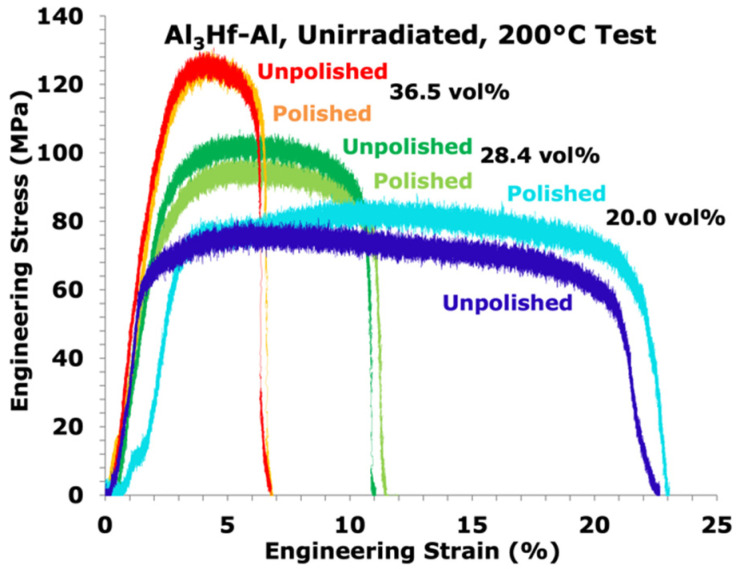
Engineering stress-strain curves of unpolished and polished unirradiated Al_3_Hf-Al specimens at 200 °C.

It was first verified that polishing the specimens for microhardness measurements would not affect the tensile properties due to potential uneven specimen cross-sectional area. Since the elevated temperature performance of this material was considered most important, a comparison test on an unpolished and polished specimen was performed for each unirradiated MMC at 200 °C. While typical test-to-test variability in tensile response was observed (shown in Figure 8), there was no consistent difference in tensile properties between polished and unpolished specimens.

**Figure 9:**
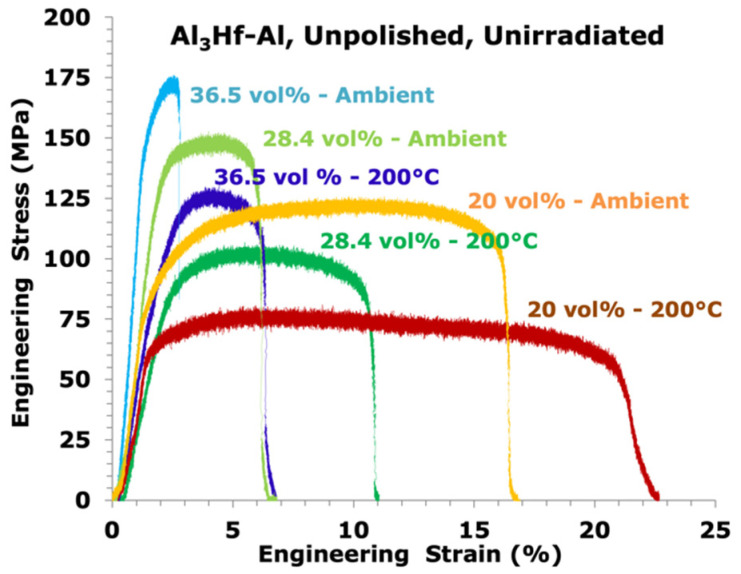
Engineering stress vs. strain curves of Al_3_Hf-Al as a function of Al_3_Hf volume fraction for the unirradiated specimens.

**Figure 10:**
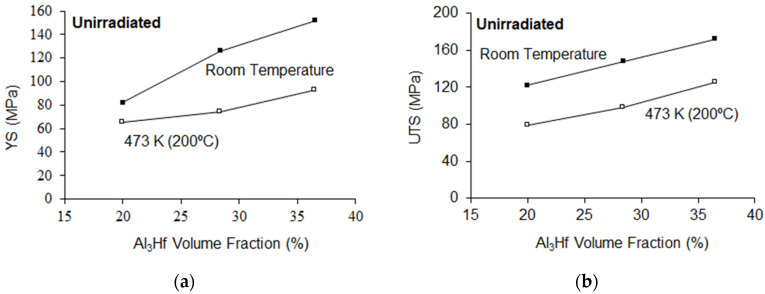
Al_3_Hf-Al samples (**a**) YS and (**b**) UTS as a function of test temperature for the unirradiated condition.

**Figure 11:**
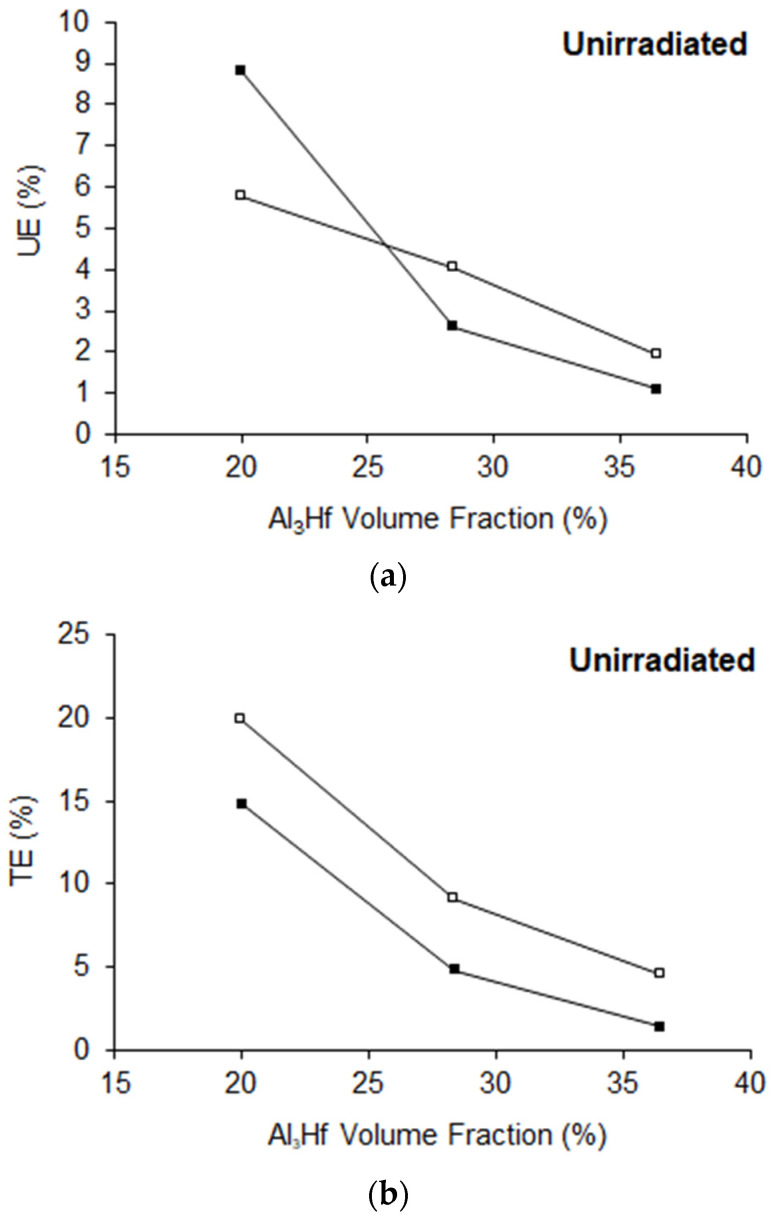
Al_3_Hf-Al samples (**a**) UE and (**b**) TE as a function of temperature for the unirradiated condition. NOTE: Filled symbol represents RT tests and unfilled symbol represents 200 °C tests.

Engineering stress versus strain curves of MMCs as a function of Al_3_Hf volume fraction and test temperature is shown in Figure 9. Tabulated values of yield strength (YS), ultimate tensile strength (UTS), uniform elongation (UE), and total elongation (TE) of the unirradiated materials as a function of Al_3_Hf volume fraction are provided in Table 6 and Table 7 for RT and 200 °C tensile tests, respectively, while plots are provided in Figure 10 and Figure 11. The relationship between YS and vol% at RT is roughly linear, just as it was for the hardness tests. UTS also increased linearly with vol% while UE and TE follow a decreasing relationship with vol%. For tests conducted at 200 °C, YS and UTS are generally below the RT values as expected. TE and UE increased with test temperature, except for the uniform elongation at 20 vol%, where there is a kink in the curve at RT. This difference may simply be due to inhomogeneity of the MMC.

While the submicron particle density within the aluminum matrix has not been counted, it is likely to be too low to act as an effective Orowan barrier to dislocation motion [37]. Instead, the primary strengthening mechanism of the unirradiated material is attributed to the intermetallic particles providing improved load carrying capacity and constraining deformation of the Al matrix [38,39,40].

**Figure 12:**
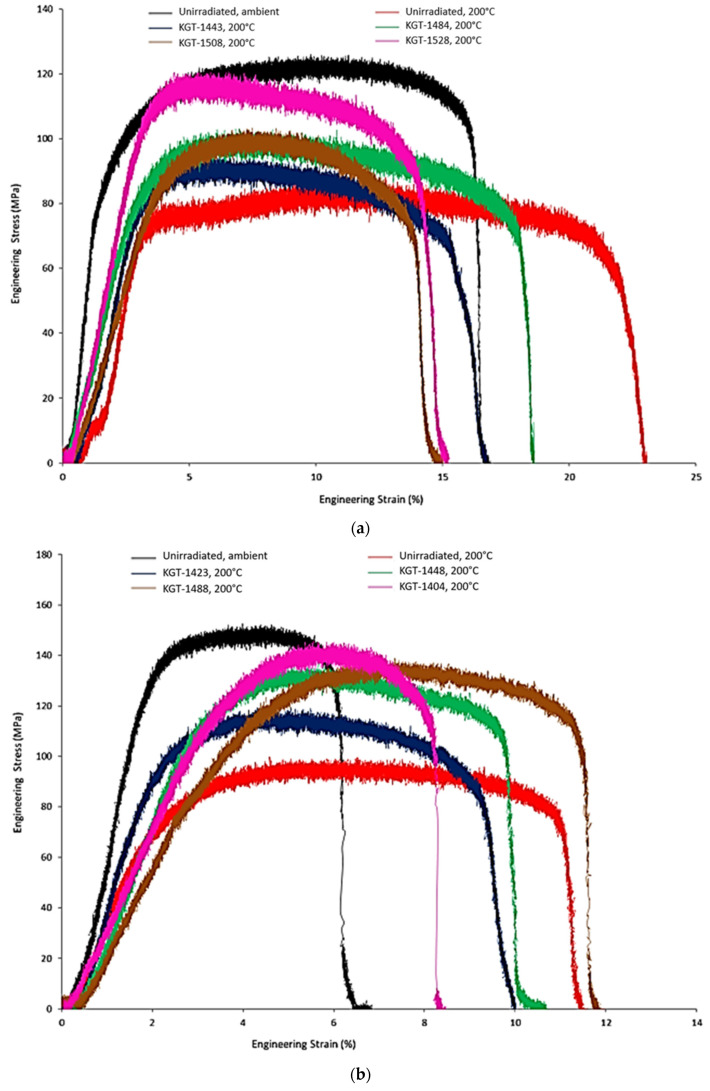

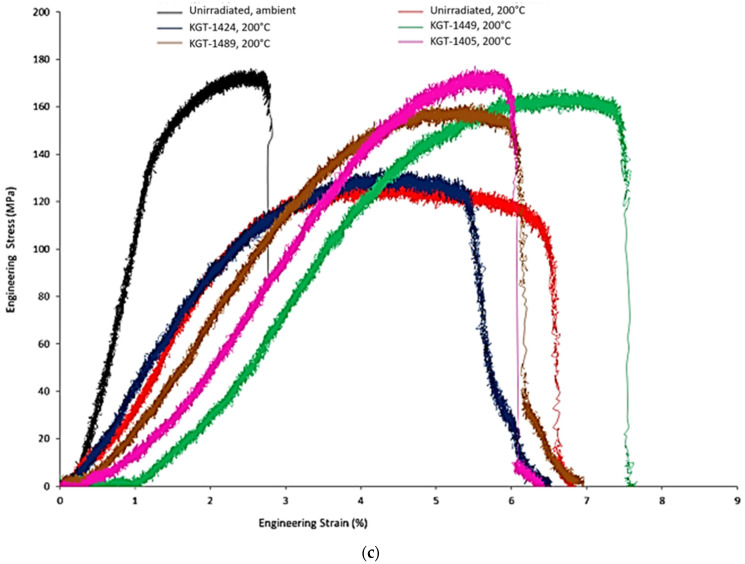
Engineering stress vs. strain curves of Al_3_Hf-Al as a function of Al_3_Hf volume fraction, test temperature and KGT identifier (**a**) 20.0 vol%, (**b**) 28.4 vol%, and (**c**) 36.5 vol%.

Table 8 and Table 9 list the tensile properties of the neutron irradiated Al_3_Hf-Al MMCs that were tested at 200 °C. The cycle average irradiation temperature and total dose over all irradiation cycles are listed. Engineering stress versus strain curves of the MMCs as a function of Al_3_Hf volume fraction, test temperature, and KGT identifier are shown in Figure 12. Tensile traces for the tests conducted at 200 °C show large strain serrations during plastic deformation that are characteristic of dynamic strain aging common in elevated temperature testing of aluminum alloys [41]. A post-yield plateau observed in the unirradiated 20.0 vol% specimen tested at 200 °C is another characteristic indicator of dynamic strain aging. The irradiated specimens exhibited a reduced dynamic strain aging response, likely due to an increase in the number of barriers to dislocation motion in the aluminum matrix that slowed the advance of dislocations independently of the solute atmospheres associated with dynamic strain aging.

**Figure 13:**
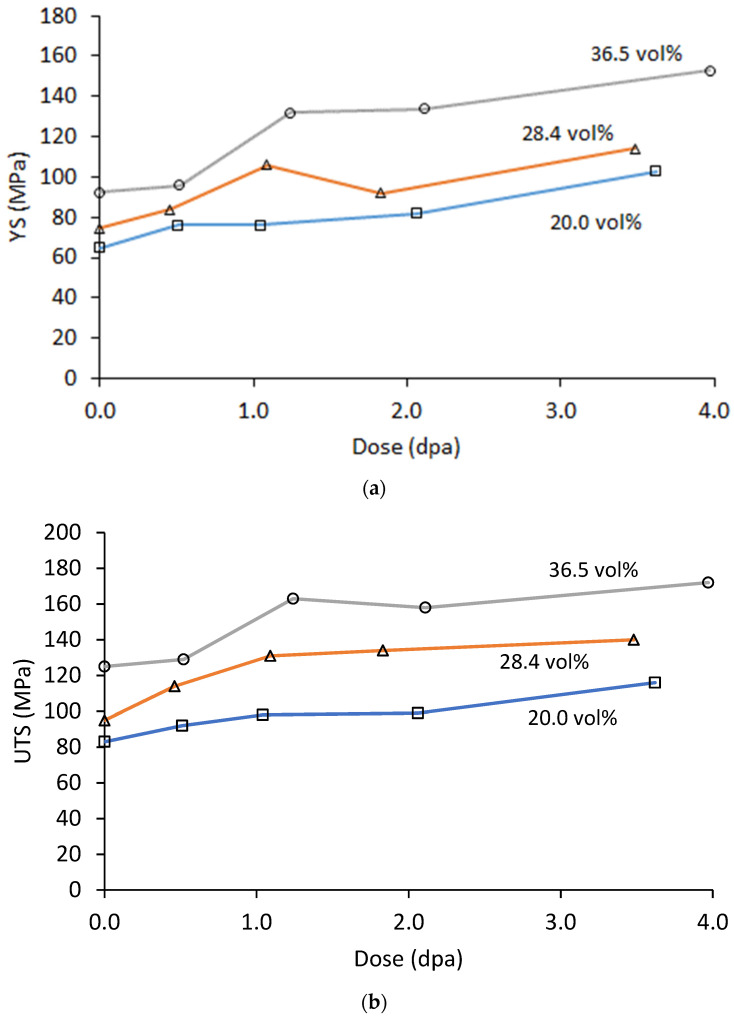
(**a**) YS and (**b**) UTS at 200 °C of the irradiated Al_3_Hf-Al materials as a function of dose.

**Figure 14:**
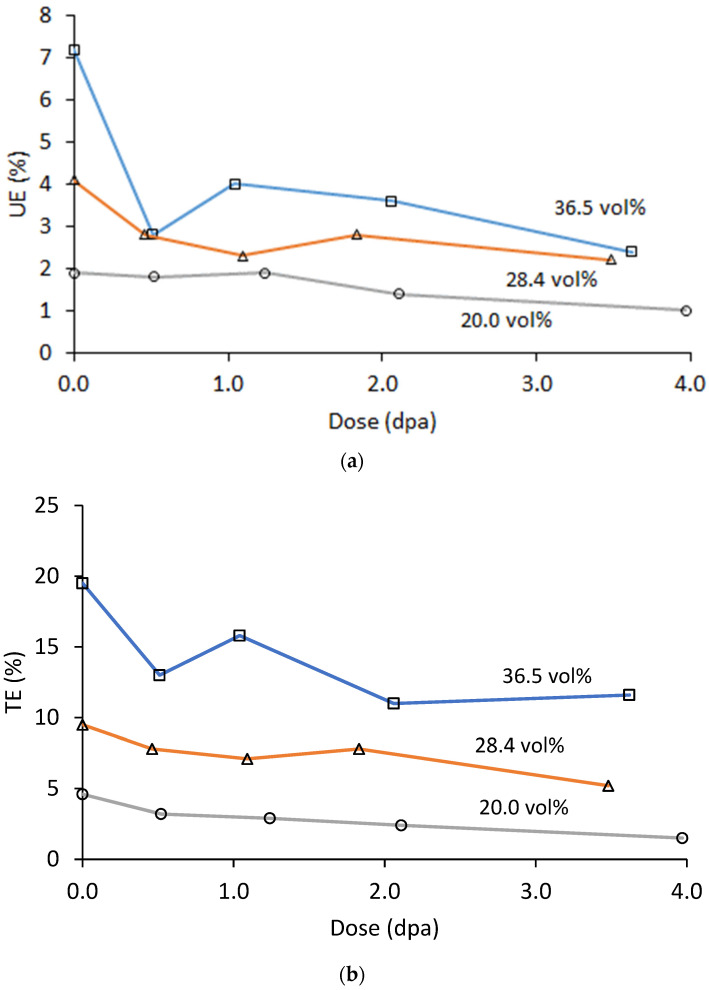
(**a**) UE and (**b**) TE at 200 °C of the irradiated Al_3_Hf-Al materials as a function of dose.

The tensile properties measured at 200 °C as a function of dose are shown in Figure 13 and Figure 14. Both YS and UTS show a generally increasing trend with dose out to the peak dose of ~3.8 dpa. Some variations in these trends are apparent and may be due to differences in irradiation temperature from the target value, or the variability could be due to inhomogeneity of the MMC. As with the hardness tests, the difference in strength (both YS and UTS) between the three materials is roughly maintained out to the peak dose, suggesting a radiation-induced hardening mechanism acting independently of the Al_3_Hf dispersion.

As often occurs, UE and TE decreased with irradiation dose [42]. The buildup of barriers to dislocation motion not only increases the strength of the material but also speeds up the work hardening process. UE for each of the three materials has nearly converged by ~3.8 dpa to a value of 1–3%, but TE shows much less convergence, with the 20.0 vol% material maintaining a TE of 10–12% at the peak dose while the 36.5 vol% material dropped from a starting value of ~4% to ~2% at peak dose. The relatively steady increase in strength and decrease in uniform elongation is typical of low to moderate temperature irradiations where thermal effects on microstructure evolution do not dominate [43].

**Figure 15:**
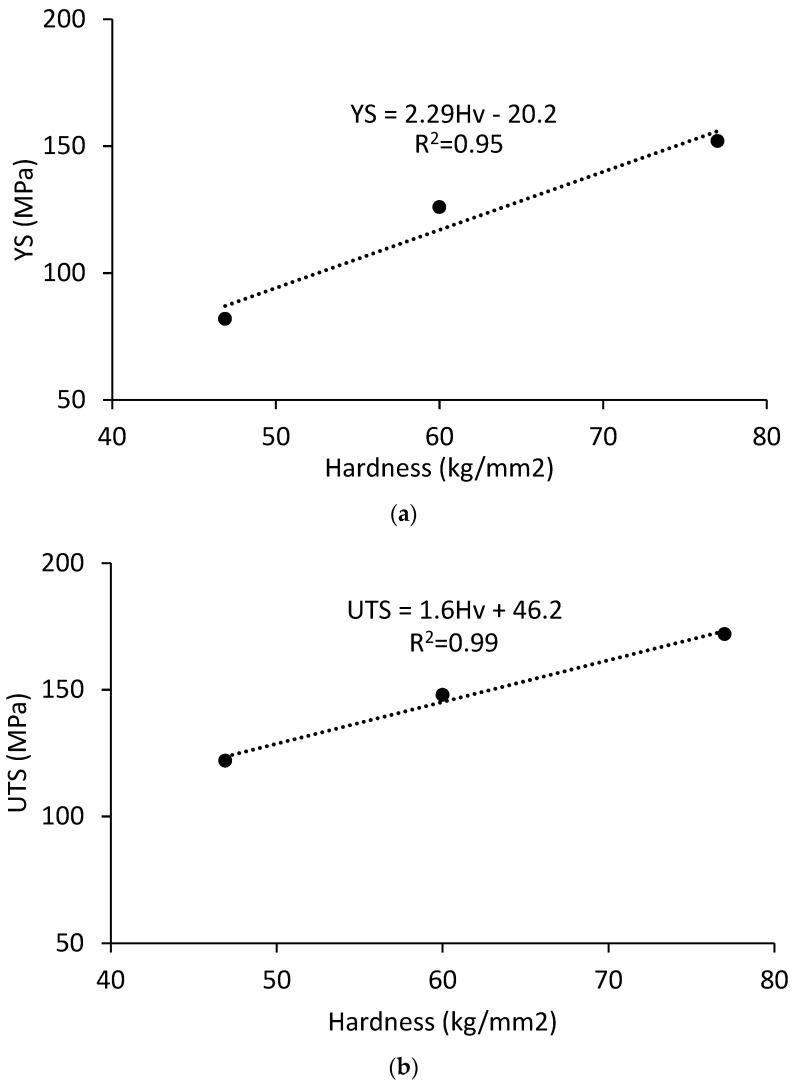
Correlations between hardness and (**a**) yield strength and (**b**) ultimate strength for the Al_3_Hf-Al MMCs at room temperature in the unirradiated condition.

RT YS and UTS of the irradiated MMCs can be measured from RT hardness by using the correlation between tensile properties and hardness measured on the unirradiated MMCs. Busby has shown that the correlation between YS and hardness for unirradiated and irradiated austenitic and ferritic steels is comparable [31], and this similarity is assumed to hold true for the aluminum MMCs studied here. A linear correlation between UTS and hardness has been obtained for unirradiated aluminum alloys [32] suggesting that it is also possible to estimate the RT UTS of the irradiated materials from hardness. Correlations between hardness and yield stress or ultimate tensile stress for the unirradiated MMCs are presented in Figure 15. It is recognized that while only three data points are available to create the correlation, the coefficient of determination (i.e., R^2^ value) for a linear fit is good.

**Figure 16:**
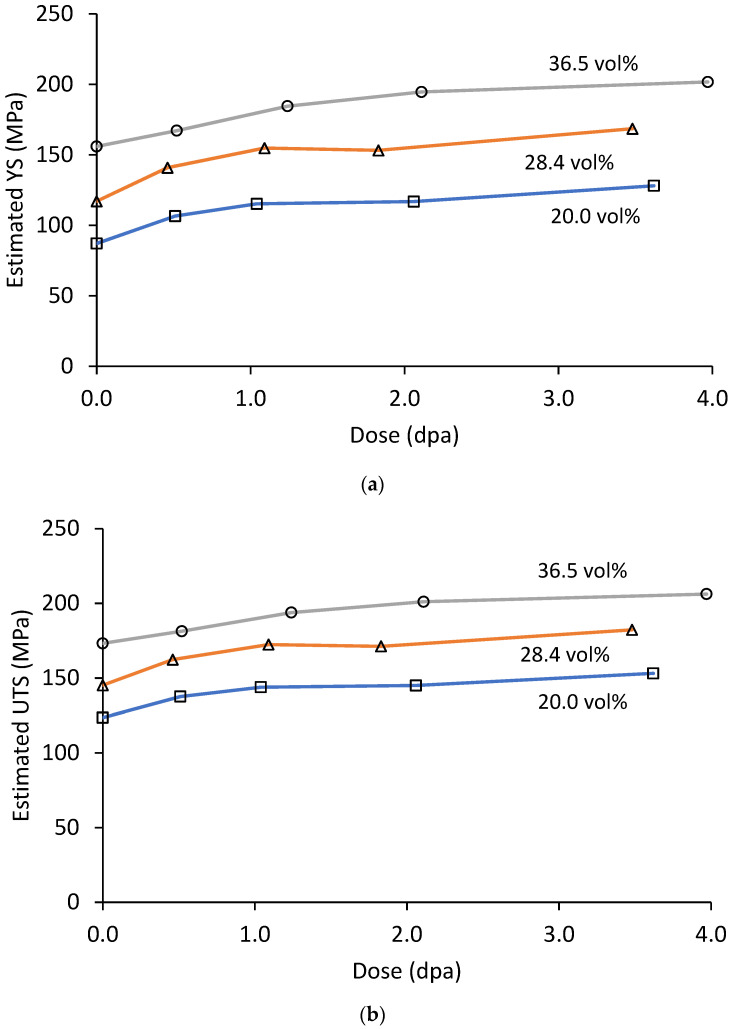
(**a**) Estimated yield strength and (**b**) estimated ultimate strength for the Al_3_Hf-Al MMCs at room temperature as a function of dose.

Estimated room temperature YS and UTS as a function of dose based on the unirradiated material correlations are presented in Figure 16. Not unexpectedly, the trends versus dose match that of the hardness data. More noteworthy is that the ratio of UTS/YS drops very close to 1.0 for the higher volume fractions. This is a strong indicator that the room temperature tensile elongation of the irradiated materials is lower than observed for the 200 °C tensile tests.

### 3.3. Thermal Expansion Measurements

**Figure 17:**
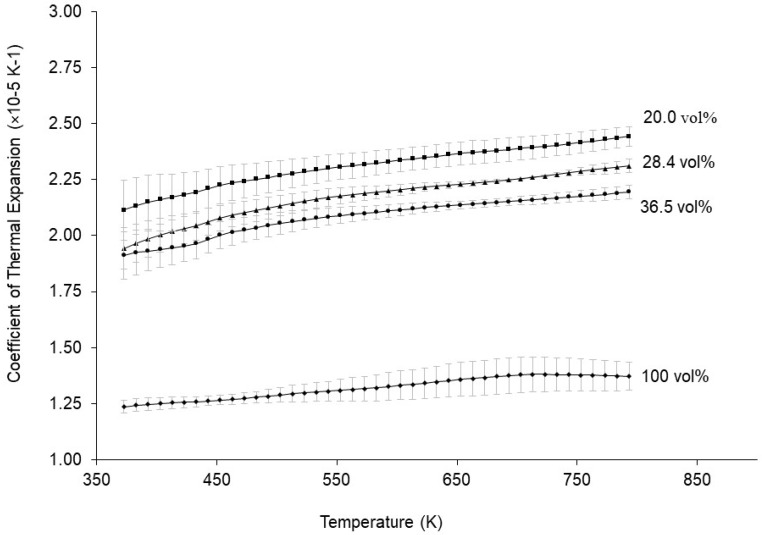
Coefficient of thermal expansion vs. temperature for the 20.0, 28.4, 36.5, and 100 vol% unirradiated materials.

Thermal expansion results of the unirradiated MMCs are shown in Figure 17. Because Al_3_Hf has a lower coefficient of linear expansion ($\alpha_{L}$) than pure aluminum, as shown by the 100 vol% curve, the observed trend of decreasing $\alpha_{L}$ with increasing Al_3_Hf volume fraction was expected based on a simple volume average. Regression analysis reveals that $\alpha_{L}$ of the unirradiated MMC can be well-approximated as a linear function of both temperature and Al_3_Hf volume fraction using the formula and coefficients shown in Table 10.

**Figure 18:**
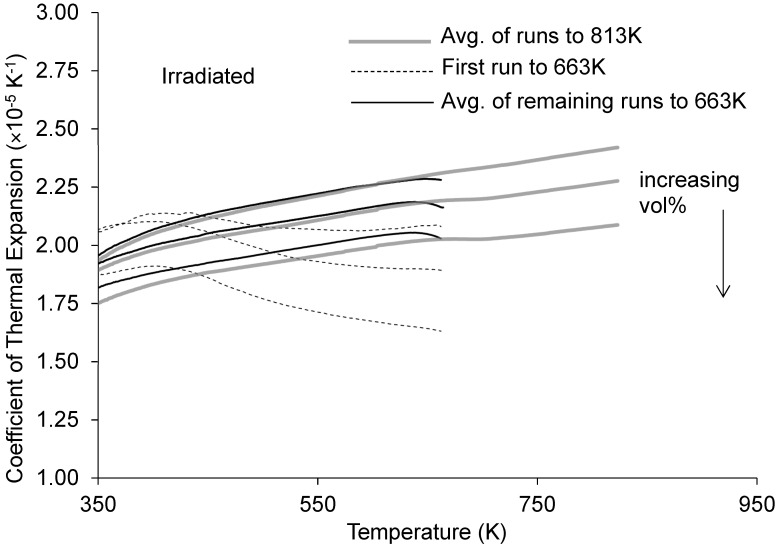
Coefficient of thermal expansion vs. temperature for the irradiated materials showing effects of annealing. Note that the 100 vol% Al_3_Hf material was not irradiated so the plot only shows three curves for the materials that were irradiated (i.e., 20, 28.4, and 36.5 vol%).

The corresponding plots for specimens irradiated for 3984.6 MWd in the ATR to a total calculated fluence of 12.02 × 10^25^ n/m^2^ are presented in Figure 18. As observed for the unirradiated materials, $\alpha_{L}$ values for the irradiated materials decrease with increasing vol%. A noteworthy feature of the data for each sample is the significant difference between the thermal expansion vs. temperature behavior measured in the first thermal expansion test compared to that observed in the remaining tests. During the first set of measurements, $\alpha_{L}\;$ increased to a maximum at ~400 K (127 °C), after which a general decreasing trend is seen. However, after the sample has been cooled to room temperature and reheated, subsequent measurements show $\alpha_{L}$ increasing nearly or all the way out to the peak observation temperature. This is true whether the sample was heated to 663 K (390 °C) or 813 K (540 °C) in these subsequent measurements, suggesting that the material is annealing at temperatures below 390 °C. A previous study found, based on exotherms observed during differential scanning calorimetry measurements, that annealing initiates at ~688 K (415 °C) for the specimens irradiated for 3984.6 MWd (3.5–3.9 dpa) [36]. However, an additional noticeable trend in Figure 18 is the further decrease in $\alpha_{L}$ observed during the runs to 813 K (540 °C) compared to the runs to 663 K (390 °C).

In comparing thermal expansion measurements of the unirradiated to the annealed irradiated materials, a maximum percentage decrease of 6.3% for the 36.5 vol% material was observed at 100 °C. While the magnitude of this decrease increases with increasing Al_3_Hf volume fraction, this is only slightly beyond the estimated 5% measurement uncertainty, and for most other data points the change in thermal expansion for the annealed irradiated materials is insignificant relative to their unirradiated states. The only significant difference was observed for $\alpha_{L}$ measured at ~660 K (387 °C) during the first measurement run, and before much annealing has occurred. This strongly suggests that the annealing process partially restores the material to its unirradiated condition.

**Figure 19:**
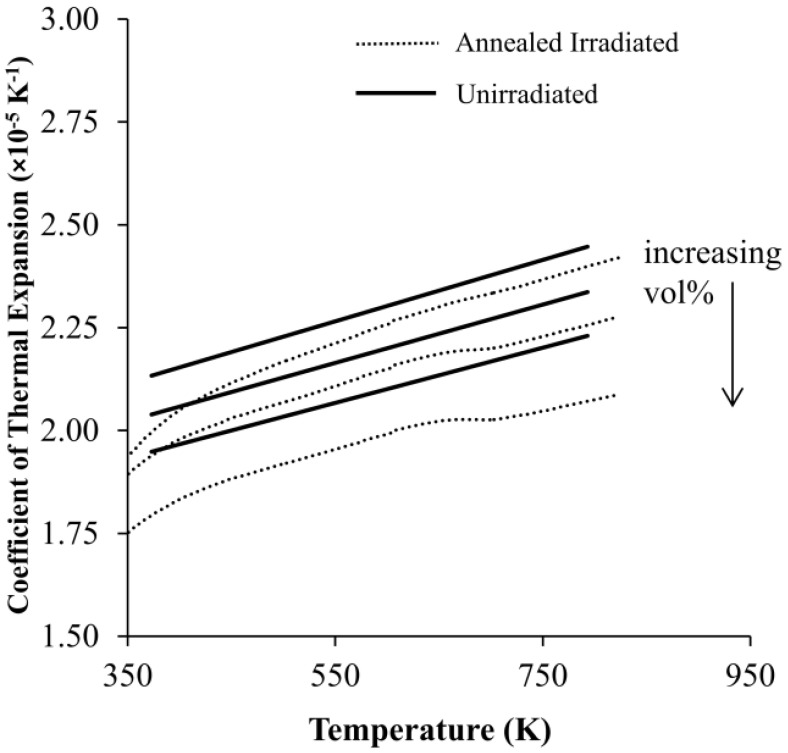
Comparison of coefficients of thermal expansion for the unirradiated materials and the annealed irradiated materials.

Figure 19 compares the fitted $\alpha_{L}$ of the unirradiated MMCs to the measured $\alpha_{L}$ of the annealed irradiated MMCs. It is readily observed that $\alpha_{L}$ is lower for the annealed irradiated specimens and that the magnitude of this change increases with the Al_3_Hf volume fraction. Regression analysis reveals that $\alpha_{L}$ of the annealed irradiated MMC can be approximated as a quadratic function of temperature using the formula and coefficients shown in Table 11.

### 3.4. Deformation Behavior and Fractography

**Figure 20:**
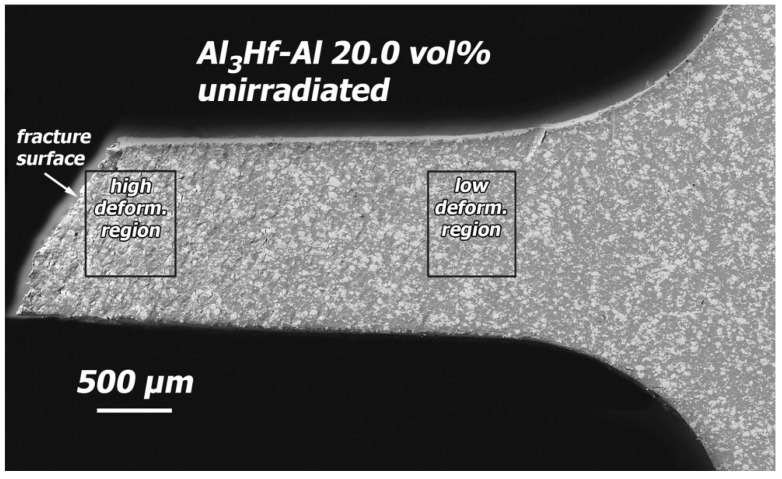
SEM image of the polished surface of an unirradiated 20 vol% specimen that has been tensile tested.

**Figure 21:**
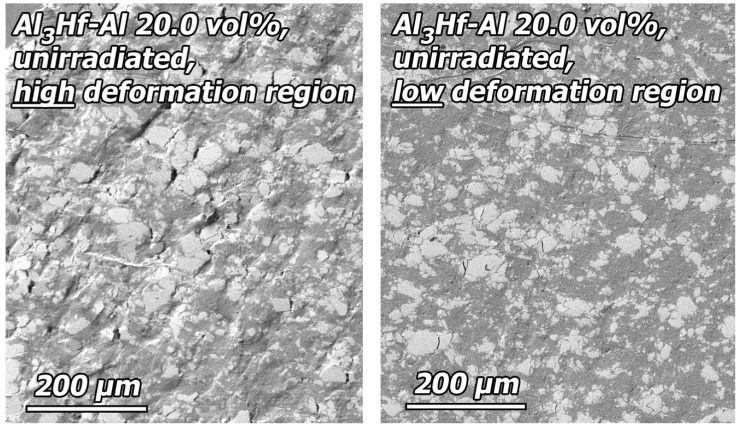
SEM image of the detail region near the fracture surface an unirradiated 20 vol% specimen that has been tensile tested.

Post-tensile test SEM images were obtained of the polished faces of 20 and 36.5 vol% specimens in the unirradiated condition and after irradiation to the highest dose. The polished faces of the specimens were used to assist in understanding the deformation behavior of the materials. Both materials exhibited similar deformation behavior, so only observations of the 20 vol% are shown. An overview SEM image of the polished surface of half of a tested unirradiated 20 vol% specimen is provided in Figure 20, showing a slanted fracture surface, typical of ductile failure. Images of the polished gauge surface at locations of higher and lower deformation (Figure 21) reveal that in regions of high deformation (up to 20% plastic strain), deformation associated with the Al_3_Hf particles is primarily accommodated by cracking, but there are also several examples of particles torn away from the matrix. Deformation bands in the Al matrix are visible and run parallel to the fracture surface. In regions of low deformation (a few percent plastic strain), cracked particles are again present, but there are no instances of particles torn away from the matrix.

**Figure 22:**
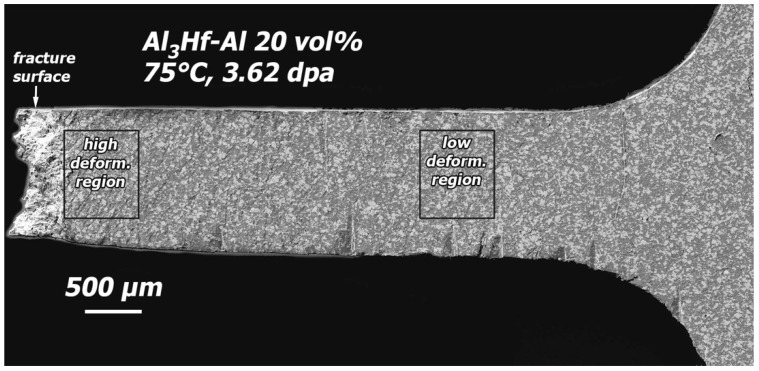
SEM image of the polished surface of the 20 vol% tensile specimen that was tensile tested after irradiation to 3.62 dpa at 75 °C (KGT-1528).

**Figure 23:**
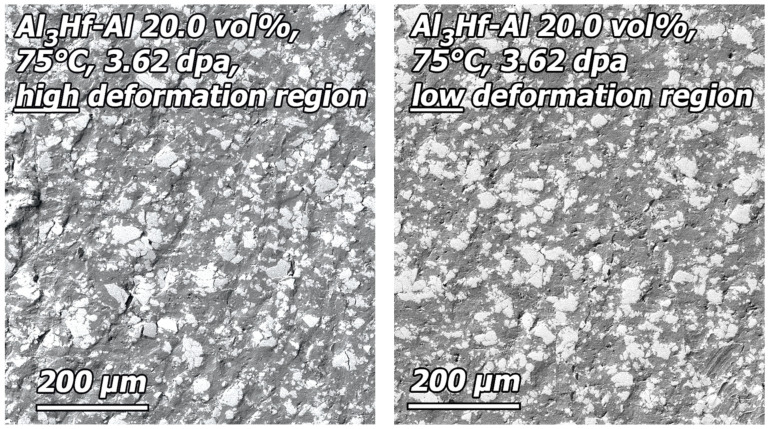
SEM image of the detail region near the fracture surface an irradiated 20 vol% specimen that has been tensile tested (KGT-1528).

Minimal changes in deformation behavior occurred after irradiation. An overview SEM image of the highest dose 20 vol% tensile specimen (KGT-1528) after tensile testing (Figure 22) shows that a lesser amount of slanted deformation occurred at the region of fracture, and the slant changed from running across the width of the specimen to across the thickness of the specimen. Examination of high and low deformation regions of the polished gauge surface (Figure 23) after testing revealed cracked particles along with some particles torn away from the matrix just as with the unirradiated specimen. Slanted deformation bands are present in both the high and low deformation regions.

**Figure 24:**
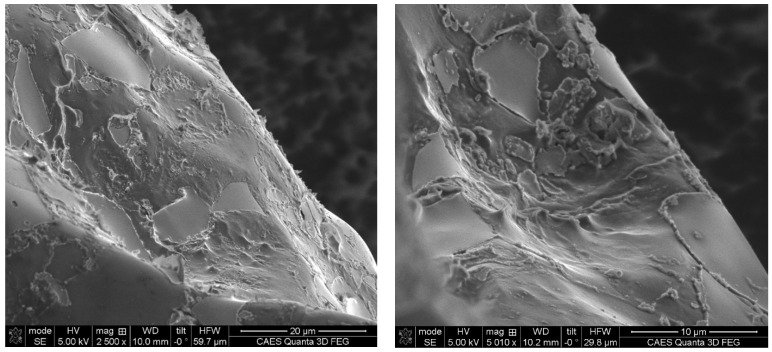
SEM images of portions of the fracture surface of the 28.4 vol% Al_3_Hf-Al irradiated specimen (KGT-1404).

Fractography was performed on KGT-1404 (e.g., the 28.4 vol% irradiated Al_3_Hf-Al material). The prevailing fracture mode at 200 °C for the irradiated material was ductile for the aluminum matrix and brittle for the intermetallic particles. From the SEM images of the fracture surface shown in Figure 24, there is no loss of cohesion between the particles and the matrix. The topography of the fracture surface is punctuated by smooth particle surfaces with tearing occurring in the ductile matrix regions.

### 3.5. Microstructural Characterization

**Figure 25:**
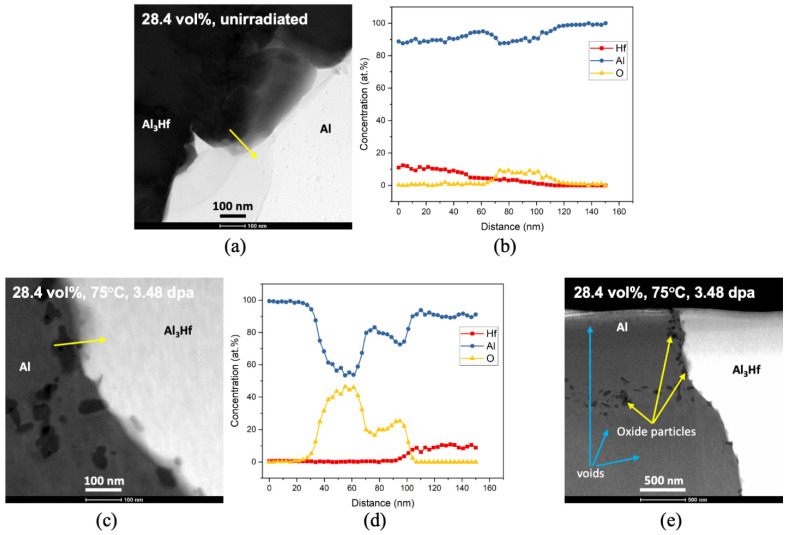
(**a**,**b**) BF STEM images and corresponding EDS linescans for unirradiated 28.4 vol% Al_3_Hf-Al (arrow indicates direction of the linescan), (**c**,**d**) A STEM Z-contrast image and corresponding EDS linescan for irradiated 28.4 vol% Al_3_Hf-Al from KGT-1404 (arrow indicates direction of the linescan), (**e**) A STEM Z-contrast image of irradiated Al_3_Hf-Al to show all the features identified.

TEM characterization was performed to understand the microstructure before and after irradiation. Figure 25a shows a bright-field STEM (BF STEM) image for unirradiated 28.4 vol% Al_3_Hf-Al. In the BF STEM image, the darker region corresponds to Al_3_Hf while the bright region corresponds to Al. Aside from the bright Al and darker Al_3_Hf regions, there is another feature with grey contrast at the phase boundary. To study the coherency at the phase boundary, an EDS linescan was performed across the phase boundary and the result is shown in Figure 25b. The grey feature contains mostly Al, with a small amount of oxygen and a trace amount of Hf.

**Figure 26:**
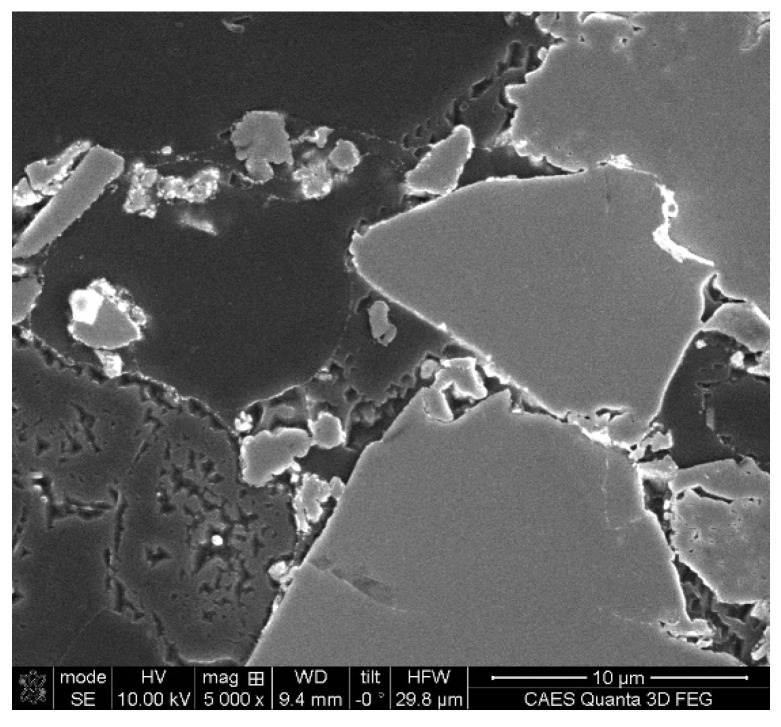
SEM image showing voids in aluminum matrix (KGT-1404).

Figure 25c shows a STEM Z-contrast image of irradiated 28.4 vol% Al_3_Hf-Al. Note that in the STEM Z-contrast image, the contrast is proportional to the average atomic number, which is opposite to what is observed in the BF STEM image. The darker region corresponds to Al while the bright region corresponds to Al_3_Hf. The irradiated Al_3_Hf-Al looks quite different compared with its unirradiated counterpart at first glance. Several particle features appear at the Al_3_Hf-Al phase boundary and the Al grain boundary. EDS analyses in Figure 25d show that these features are enriched with Al and O with a stoichiometry ratio of almost 1:1, which indicates that they are likely to be AlO particles. In addition to these oxide particles, there are voids within the Al matrix after irradiation as shown in Figure 25e and Figure 26.

Comparing the unirradiated and irradiated Al_3_Hf-Al samples, the unirradiated Al_3_Hf-Al only exhibits a small amount of chemical segregation, with oxygen concentrated at the phase boundary. Irradiation further induced it to form an oxide particle with Al. These oxide particles and voids can serve as obstacles to pin the dislocation movement, therefore causing hardening and ductility reduction of the material. While the barrier hardening coefficient of these large voids and oxide particles is known to be ~1 [44], without detailed information about the size and number density of these features, it is impossible to use a barrier hardening model to estimate whether these are largely responsible for the observed YS increase. Additional TEM examinations and quantitative measurements of feature populations would be needed.

## 4. Summary

The effects of neutron irradiation on the hardness, strength, ductility, and coefficient of thermal expansion on an Al_3_Hf-Al MMC were reported. Knowledge of these properties is needed to effectively develop absorber blocks to facilitate fast flux testing of fuels and materials in existing light water reactors. The key findings are summarized here:

Microhardness testing of unirradiated Al_3_Hf-Al MMC materials at room temperature showed a roughly linear trend between hardness and volume fraction with an approximate 1.6× increase in the hardness between 20.0 vol% and 36.5 vol% specimens.

Tensile testing of unirradiated materials showed that the relationship between YS and volume fraction at room temperature is roughly linear, just as it was for the hardness tests. UTS also increased linearly with vol% while UE and TE followed a decreasing relationship with vol%. For tests conducted at 200 °C, YS and UTS were generally below the room temperature values as expected. TE and UE increased with test temperature, except for the uniform elongation at 20 vol%, where there is a kink in the curve at room temperature. This difference may simply be due to test-to-test variability.

The primary strengthening mechanism of the unirradiated Al_3_Hf-Al MMC is attributed to the intermetallic particles providing improved load carrying capacity and providing greater constraint against deformation for the Al matrix.

Microhardness of the irradiated Al_3_Hf-Al MMC tested at room temperature increased with dose and appeared to trend towards a plateau value by ~3.5 dpa. In each of the three volume fraction MMCs, nearly the same amount of irradiation hardening occurred, suggesting that the hardening is not strongly tied to alteration of the Al_3_Hf dispersion but instead may be due to matrix hardening effects that operate independently of the dispersion.

Tensile testing of irradiated materials at 200 °C showed that the difference in strength (both YS and UTS) between the three vol% materials is roughly maintained out to the peak dose, suggesting a radiation-induced hardening mechanism acting independently of the Al_3_Hf dispersion, similar to the room temperature microhardness test results. UE and TE decreased with irradiation dose for all three vol% materials.

SEM performed on the fracture surface of irradiated 28.4 vol% Al_3_Hf-Al MMC showed evidence of brittle fracture of the particles and ductile tearing in the matrix regions with some instances of particles tearing away from the matrix.

Not surprisingly, the 36.5 vol% material exhibited the lowest starting ductility and lowest ductility after irradiation with the uniform elongation dropping to ~1% at ~3.8 dpa. Ductility at room temperature would be even lower, calling into question whether the 36.5 vol% could be used for the absorber block. The 28.4 vol% material with 2% UE and 5% TE after ~3.5 dpa is more ductile.

Thermal expansion is a key thermal property that is important to the design of gaps and clearances surrounding the absorber block. The dilatometry results show decreasing $\alpha_{L}$ with increasing Al_3_Hf volume fraction. The data has been regressed into equations approximated as a quadratic function of temperature for the thermal expansion of the unirradiated and the annealed irradiated material. The $\alpha_{L}$ of the unirradiated material is higher than that of the irradiated material, although the annealing process partially restores the material to its unirradiated condition and thermal expansion behavior.

EDS linescans reveal the reaction of oxygen at the phase boundary between the particles and the matrix. The role of oxygen in forming AlO at the phase boundary is more significant for the irradiated than the unirradiated material.

## Figures and Tables

**Table 1:** As-run irradiation conditions of Al_3_Hf-Al tensile specimens [27].

Sample ID	Al_3_Hf (vol%)	MWd	Fluence (×10^25^ n/m^2^)	Est. Avg. Irr. Temp. (°C)	Dose (dpa)
KGT-1443	20.0	800.6	1.382	72	0.51
KGT-1423	28.4	800.6	1.382	70	0.46
KGT-1424	36.5	800.6	1.382	70	0.52
KGT-1484	20.0	1965.5	2.403	66	1.04
KGT-1448	28.4	1965.5	2.403	67	1.09
KGT-1449	36.5	1965.5	2.403	67	1.24
KGT-1508	20.0	3184.0	9.33	70	2.06
KGT-1488	28.4	3184.0	9.33	69	1.83
KGT-1489	36.5	3184.0	9.33	69	2.11
KGT-1528	20.0	3984.6	12.02	75	3.62
KGT-1404	28.4	3984.6	12.02	75	3.48
KGT-1405	36.5	3984.6	12.02	74	3.97

**Table 2:** Density and weight percent of elemental content as a function of Al_3_Hf vol%.

	20 vol% Al_3_Hf	28.4 vol% Al_3_Hf	36.5 vol% Al_3_Hf	100% Al_3_Hf
**Density** (kg m^−3^)	3.43	3.74	3.95	6.03
**Element**	**Elemental Composition (wt%)**			
Al	74.76	67.04	60.7	31.2
Zr	0.885	0.885	0.885	0.885
Hf	24.36	32.08	38.42	67.92

**Table 3:** Irradiation conditions for thermal expansion specimens [27,28].

Specimen ID	Al_3_Hf vol%	Irr. Temp. (°C)	Dose (dpa)
KGT-1399	20.0	84	3.63
KGT-1536	28.4	125	3.56
KGT-1544	36.5	84	3.55

**Table 4:** Vickers microhardness values of Al_3_Hf-Al samples as a function of dose and volume fraction of Al_3_Hf.

Specimens	Dose (dpa)	Avg. Hardness (kg/mm^2^)	Hardness Std. Dev. (kg/mm^2^)	Avg. Indent Size (µm)
**20 vol%**				
unirradiated	0	46.9	2.6	140.7
KGT-1443	0.51	55.4	2.5	129.5
KGT-1484	1.04	59.2	3.9	124.9
KGT-1508	2.06	59.9	4.4	124.6
KGT-1528	3.62	64.8	4.7	119.7
**28.4 vol%**				
unirradiated	0	60.0	4.9	124.1
KGT-1423	0.46	70.4	5.7	115.4
KGT-1448	1.09	76.5	6.9	111.4
KGT-1488	1.83	75.8	6.6	111.2
KGT-1404	3.48	82.5	7.0	106.7
**36.5 vol%**				
unirradiated	0	77.0	10.4	111.0
KGT-1424	0.52	81.9	4.6	106.7
KGT-1449	1.24	89.5	7.1	101.8
KGT-1489	2.11	93.9	10.0	99.7
KGT-1405	3.97	97.0	8.7	98.2

**Table 5:** Measured nanohardness for an unirradiated and irradiated specimen.

Type of Specimen	Hardness (GPa)
Unirradiated (36.5 vol%)	7.6 ± 0.8
Irradiated (KGT-1404; 28.5 vol%)	8.0 ± 0.3

**Table 6:** Room temperature tensile properties of the unirradiated Al_3_Hf-Al samples.

Al_3_Hf vol%	Condition	Test Temp. (°C)	0.2% Offset YS (MPa)	UTS (MPa)	UTS/YS Ratio	UE (%)	TE (%)
20.0	Unpolished	Ambient	82	122	1.49	8.8	14.8
28.4	Unpolished	Ambient	126	148	1.17	2.6	4.8
36.5	Unpolished	Ambient	152	172	1.13	1.1	1.4

**Table 7:** Tensile properties of the unirradiated Al_3_Hf-Al samples at 200 °C.

Al_3_Hf vol%	Condition	Test Temp. (°C)	0.2% Offset YS (MPa)	UTS (MPa)	UTS/YS Ratio	UE (%)	TE (%)
20.0	Unpolished	200	62	76	1.23	4.3	20.3
20.0	Polished	200	68	83	1.22	7.2	19.5
**20.0 vol% Average**			**65**	**79.5**	**1.225**	**5.8**	**19.9**
28.4	Unpolished	200	83	102	1.23	4.0	8.6
28.4	Polished	200	66	95	1.44	4.1	9.5
**28.4 vol% Average**			**74.5**	**98.5**	**1.34**	**4.05**	**9.05**
36.5	Unpolished	200	93	125	1.34	1.9	4.4
36.5	Polished	200	92	125	1.36	1.9	4.6
**36.5 vol% Average**			**92.5**	**125**	**1.35**	**1.9**	**4.5**

**Table 8:** Tensile properties of the irradiated Al_3_Hf-Al samples tested at 200 °C.

Specimen Identifier	Al_3_Hf vol%	Irr. Temp. (°C)	Dose (dpa)	Test Temp. (°C)	0.2% offset YS (MPa)	UTS (MPa)	UTS/YS	UE (%)	TE (%)
B3 (unirr)	20.0	N/A	0	200	65	83	1.22	7.2	19.5
KGT-1443	20.0	72	0.51	200	76	92	1.21	2.8	13.0
KGT-1484	20.0	66	1.04	200	76	98	1.29	4.0	15.8
KGT-1508	20.0	70	2.06	200	82	99	1.21	3.6	11.0
KGT-1528	20.0	75	3.62	200	103	116	1.13	2.4	11.6
B2 (unirr)	28.4	N/A	0	200	74.5	95	1.44	4.1	9.5
KGT-1423	28.4	70	0.46	200	84	114	1.36	2.8	7.8
KGT-1448	28.4	67	1.09	200	106	131	1.24	2.3	7.1
KGT-1488	28.4	69	1.83	200	92	134	1.34	2.8	7.8
KGT-1404	28.4	75	3.48	200	114	140	1.23	2.2	5.2
B1 (unirr)	36.5	N/A	0	200	92.5	125	1.36	1.9	4.6
KGT-1424	36.5	70	0.52	200	96	129	1.34	1.8	3.2
KGT-1449	36.5	67	1.24	200	132	163	1.23	1.9	2.9
KGT-1489	36.5	69	2.11	200	134	158	1.18	1.4	2.4
KGT-1405	36.5	74	3.97	200	153	172	1.12	1.0	1.5

Note: all specimens were polished on one side to enable microhardness testing on the tabs.

**Table 9:** Changes in mechanical properties of Al_3_Hf-Al as a result of irradiation.

Specimen Identifier	Al_3_Hf vol%	Dose (dpa)	Test Temp. (°C)	Percent Change After Irradiation			
				0.2% offset YS	UTS	UE	TE
B3 (unirradiated)	20	0	200	0	0	0	0
KGT-1443		0.51		17	11	−61	−33
KGT-1484		1.04		17	18	−44	−19
KGT-1508		2.06		26	19	−50	−44
KGT-1528		3.62		58	40	−67	−41
B2 (unirradiated)	28.4	0	200	0	0	0	0
KGT-1423		0.46		13	20	−32	−18
KGT-1448		1.09		42	38	−44	−25
KGT-1488		1.83		23	41	−32	−18
KGT-1404		3.48		53	47	−46	−45
B1 (unirradiated)	36.5	0	200	0	0	0	0
KGT-1424		0.52		4	3	−5	−30
KGT-1449		1.24		43	30	0	−37
KGT-1489		2.11		45	26	−26	−48
KGT-1405		3.97		65	38	−47	−67

**Table 10:** Regression results for measured linear coefficients of thermal expansion for the unirradiated materials.

$\mathrm{Form}:\;\alpha_{L}\left(f,T\right)=a_{1}f+a_{2}T+a_{3}fT+a_{4}$ $\;\mathrm{with}\;\mathrm{units}\;\alpha_{L}\left[=\right]\times 10^{-5}\;K^{-1},\;T\left[=\right]K,\;f\left[=\mathrm{vol}\%\right]$			
$a_{1}$	$a_{2}$	$a_{3}$	$a_{4}$
−9.499 × 10^−3^	8.375 × 10^−4^	−4.560 × 10^−6^	2.045

**Table 11:** Regression results for measured linear coefficients of thermal expansion for annealed irradiated material.

$\alpha_{L}\left(T\right)=b_{1}T^{2}+b_{2}T+b_{3}$ $\;\mathrm{with}\;\mathrm{units}\;\alpha_{L}\left[=\right]\times 10^{-5}\;K^{-1},\;T\left[=\right]K$			
Al_3_Hf vol%	$b_{1}\;\left(\times 10^{-6}\right)$	$b_{2}\;\left(\times 10^{-3}\right)$	$b_{3}$
20.0	−1.890	3.192	1.039
28.4	−1.541	2.583	1.163
36.5	−1.882	2.891	0.9447

## Data Availability

Research data has been included in this publication or is available in the cited references.
